# No evidence for a causal link between *Helicobacter pylori* infection and nonalcoholic fatty liver disease: A bidirectional Mendelian randomization study

**DOI:** 10.3389/fmicb.2022.1018322

**Published:** 2022-11-03

**Authors:** Yuwei Liu, Hongqin Xu, ZiHan Zhao, Yutong Dong, Xiaomei Wang, Junqi Niu

**Affiliations:** ^1^Department of Hepatology, Center of Infectious Diseases and Pathogen Biology, The First Hospital of Jilin University, Changchun, China; ^2^Key Laboratory of Zoonosis Research, Ministry of Education, The First Hospital of Jilin University, Changchun, China; ^3^Division of Thyroid Surgery, China-Japan Union Hospital of Jilin University, Changchun, China

**Keywords:** causal effects, mendelian randomization, nonalcoholic fatty liver disease, *Helicobacter pylori*, null association

## Abstract

Although clinical studies have shown the possible relationship between *Helicobacter pylori* (*H. pylori*) infection and the development of nonalcoholic fatty liver disease (NAFLD), their causal relationship is still unknown. This bidirectional Mendelian randomization (MR) study aimed to investigate the causal link between *H. pylor*i infection and NAFLD. Two previously reported genetic variants SNPs rs10004195 and rs368433 were used as the instrumental variables (IVs) of *H. pylori* infection. The genetic variants of NAFLD were extracted from the largest genome-wide association study (GWAS) summary data with 1,483 cases and 17,781 controls. The exposure and outcome data were obtained from the publicly available GWAS dataset. Then, a bidirectional MR was carried out to evaluate the causal relationship between *H. pylori* infection and NAFLD. In addition, the GWAS data were also collected to explore the causal relationship between *H. pylori* infection and relevant clinical traits of NAFLD, including triglycerides, low-density lipoprotein cholesterol (LDL-C), high-density lipoprotein cholesterol (HDL-C), fasting blood glucose (FBG), and body mass index (BMI). Genetically predicted *H. pylori* infection showed no association with NAFLD both in FinnGen GWAS (OR, 1.048; 95% CI, 0.778–1.411; value of *p* = 0.759) and the GWAS conducted by Anstee (OR, 0.775; 95% CI, 0.475–1.265; value of *p* = 0.308). An inverse MR showed no causal effect of NAFLD on *H*. *pylori* infection (OR,0.978;95% CI, 0.909–1.052; value of *p* = 0.543). No significant associations were observed between *H*. *pylori* infection and the levels of triglycerides, LDL-C, HDL-C, or FBG, while *H. pylori* infection was associated with an increase in BMI. These results indicated that there was no genetic evidence for a causal link between *H. pylori* and NAFLD, suggesting that the eradication or prevention of *H. pylori* infection might not benefit NAFLD and vice versa.

## Introduction

Nonalcoholic fatty liver disease (NAFLD) is a common liver disorder characterized by liver steatosis which is considered the manifestation of metabolic syndrome in the liver (Hamaguchi et al., 2005). Approximately, 10 to 30% of patients with NAFLD may eventually develop nonalcoholic steatohepatitis (NASH), which can result in cirrhosis, hepatocellular carcinoma, and liver failure (Yu et al., 2018; Duell et al., 2022). Currently, the prevalence rates for NAFLD are gradually increasing, imposing serious economic and societal burdens. *Helicobacter pylori* (*H. pylori*) is a kind of gram-negative bacterium. It can selectively colonize the human gastric epithelium (Liu et al., 2018). *H. pylori* infection is common, with approximately, 60% of the global human population infected (Hooi et al., 2017). Previous studies have reported that *H. pylori* infection may promote insulin resistance, increase inflammatory cytokine production, and stimulate white adipose tissue to activate the related signaling pathways, which could contribute to NAFLD (Cheng et al., 2017). One animal study has shown elevated liver function and increased metabolic indexes in *H. pylori* infection mice models with high fat diet (He et al., 2018).

Several observational studies found that the prevalence of NAFLD is common in patients with *H. pylori* infection accounting for approximately, 33–47% (Kang et al., 2018; Jiang et al., 2019; Abo-Amer et al., 2020). Some meta-analysis studies also indicated that *H. pylori* infection could increase the incidence rate of NAFLD. However, other retrospective studies came out with the opposite conclusion that *H. pylori* infection is not an independent risk factor for NAFLD (Okushin et al., 2015; Alvarez et al., 2020; Han et al., 2021). In addition, it should be noted that all the available evidence is based on observational studies, which have obvious limitations such as unmeasured or imprecisely measured confounders, reverse causation, and other sources of bias. There is a paucity of definitive evidence on the causal link between *H. pylori* infection and NAFLD, which is important for the prevention or treatment of NAFLD through *H. pylori* eradication.

Mendelian randomization (MR) is an epidemiological analytic method to strengthen causal inference. The MR design utilizes genetic variants as instrument variables (IVs) for the exposure of interest, usually single nucleotide polymorphisms (SNPs), which are randomly distributed and unaffected by environmental factors and other cofounders (Davey Smith and Hemani, 2014). Thus, the MR design can rigorously account for the causal relationships between complex disorders. With the accumulation of genome-wide association studies (GWASs) and the availability of large-scale GWAS data, two-sample MR design is becoming more accessible and increasingly widespread. In this study, we first performed a two-sample MR analysis to predict *H. pylori* infection and assess its association with NAFLD in two independent, population-scale GWAS data for NAFLD. In addition, we treated the incidence of NAFLD as the exposure to explore reverse causation between *H. pylori* infection and NAFLD, hoping to clarify their causal relationship and provide useful advice for clinical practice.

## Materials and Methods

### Mendelian randomization design

**Figure 1:**
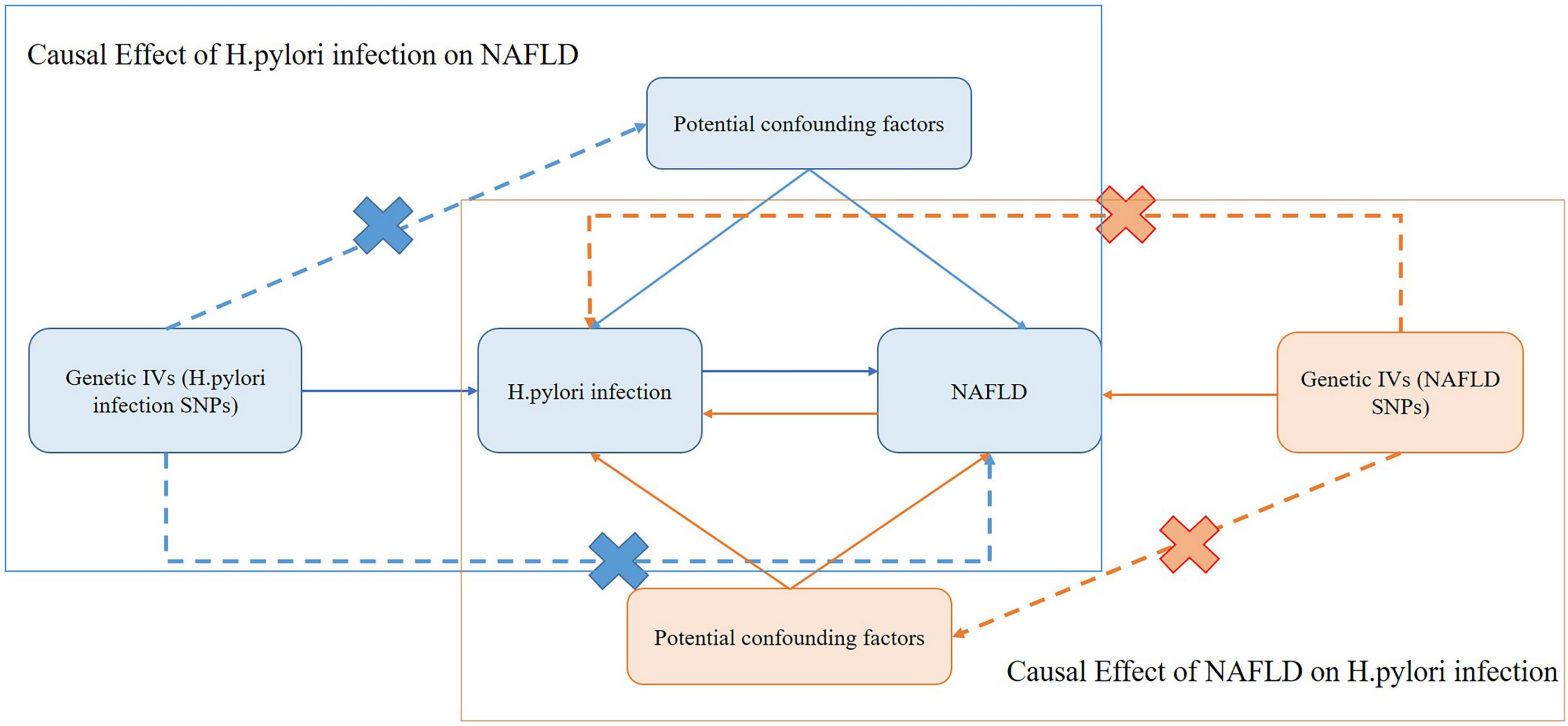
Schematic representation of the bidirectional MR study on the causal relationship between *H. pylori* infection and NAFLD. IVs, instrument variants; *H. pylori*, *Helicobacter pylori*; SNP, single-nucleotide polymorphisms; NAFLD, nonalcoholic fatty liver disease.

The framework of the current MR study was described in Figure 1. In this current study, we used genetic variants as IV for the MR analysis. The assumed validity of our MR study was based on the following three core assumptions: (1) relevance assumption: the genetic variants are strongly associated with the exposure; (2) independence assumption: the genetic variants are not associated with any confounders that might mediate ways from exposure to outcome; and (3) exclusion-restriction assumption: the genetic variants affect the outcome only possible *via* the exposure (Emdin et al., 2017).

### Data sources description

The genetic association of NAFLD was derived from two independent GWAS data; one was obtained from a recently published study composed of 1,483 European cases and 17,781 European controls (Anstee et al., 2020), and the other was composed of 894 European cases and 217,898 European controls; it was downloaded from the GWAS data sources on the FinnGen database, which is available at https://www.fifinngen.fifi/en. The NAFLD in the GWAS conducted by Anstee et al. (2020) was definitively diagnosed by histopathology after liver biopsy, while the NAFLD in the FinnGen GWAS was diagnosed according to electronic medical records. The GWAS summary data of *H. pylori* infection were obtained from the public data that had been assembled in the European Bioinformatics Institute (EBI) database at https://gwas.mrcieu.ac.uk/datasets/ieu-b-4905/, which included 1,058 European cases and 3,625 European controls. In addition, the GWAS data were also collected to investigate the causal effect between *H. pylori* infection and the relevant clinical traits of NAFLD, including triglycerides, low-density lipoprotein cholesterol (LDL-C), high-density lipoprotein cholesterol (HDL-C), fasting blood glucose (FBG), and body mass index (BMI). The GWAS summary statistics of lipid traits, including triglycerides, LDL-C, and HDL-C levels, were obtained from the United Kingdom Biobank database (Richardson et al., 2020). The GWAS summary statistics of FBG were obtained from EBI database (Manning et al., 2012), and the GWAS statistics of BMI were obtained from MRC Integrative Epidemiology Unit (MRC-IEC) database.^1^ Each GWAS was approved by corresponding Ethics Committees. The details of the GWAS data included in this study were shown in Table 1.

**Table 1:** Details of the studies included in the Mendelian randomization analyses.

Phenotype	Consortium or author	Ethnicity	Sample size	Year	Number of SNPs	Web source
NAFLD	Anstee et al	European	1,483 cases and 17,781 controls	2020	7,411,923	https://www.ebi.ac.uk/gwas
NAFLD	FinnGen study	European	894 cases and 217,898 controls	2021	16,380,466	https://gwas.mrcieu.ac.uk/datasets/finn-b-NAFLD/
*H. polyri* infection	EBI	European	1,058 cases and 3,625 controls	2021	7,247,045	https://gwas.mrcieu.ac.uk/datasets/ieu-b-4905/
Triglycerides	UK Biobank	European	441,016 participants	2020	12,321,875	https://gwas.mrcieu.ac.uk/datasets/ieu-b-111/
LDL-C	UK Biobank	European	440,546 participants	2020	12,321,875	https://gwas.mrcieu.ac.uk/datasets/ieu-b-110/
HDL-C	UK Biobank	European	403,943 participants	2020	12,321,875	https://gwas.mrcieu.ac.uk/datasets/ieu-b-109/
FBG	EBI	European	58,074 participants	2012	2,599,409	https://gwas.mrcieu.ac.uk/datasets/ebi-a-GCST005186/
BMI	MRC-IEU	European	454,884 participants	2018	9,851,867	https://gwas.mrcieu.ac.uk/datasets/ukb-b-2303/

SNP, single-nucleotide polymorphisms; NAFLD, nonalcoholic fatty liver disease; *H*. *pylori*, *Helicobacter pylori*; EBI, European Bioinformatics Institute; LDL-C, low density lipoprotein cholesterol; HDL-C, high density lipoprotein cholesterol; FBG, fasting blood glucose; BMI, body mass index.

### Selection of genetic instrumental variables for *Helicobacter pylori* infection

Genetic IVs can be acquired *via* two ways, one is from previous literature and the other is directly from the GWAS summary statistics. The genetic IVs of *H. pylori* infection were obtained from a previous study conducted by Mayerle et al. (2013). The toll-like receptor 1 (TLR1) gene SNP rs10004195 at 4p14 and the Fc gamma RIIA (FCGR2A) gene SNP rs368433 at 1q23.3 have been identified as the genetic variants for *H pylori* seroprevalence with the strongest association strength. The A allele of TLR1 has been reported to increase the risk of *H. pylori* infection (Tang et al., 2015; Kalkanli Tas et al., 2020), while FCGR polymorphisms have been implicated in persistent bacterial infections including *H. pylori* (Corcoran and Byrne, 2004). Furthermore, to examine the strength of the allele scores as instruments, the F statistic for each SNP was approximated from the following equation:


$$F=\frac{N-K-1}{K}\times \frac{R^{2}}{1-R^{2}}$$

where, N is the sample size of the exposure dataset, K is the number of SNPs, and *R*^2^ represents the proportion of the variation explained by IVs (Burgess and Thompson, 2011). The F statistic of the two SNPs was greater than 30, as shown in Table 2. Thus, the two SNPs were utilized as the IVs of *H. pylori* infection for subsequent analysis.

**Table 2:** Instrumental SNPs of *H. pylori* infection and F statistics.

Instrumental SNP	Effect allele	Other allele	Gene	EAF	BETA	SE	*P*	*F*
rs10004195	A	T	TLR1	0.25	0.3576744	0.04048331	1.00E-18	478.17
rs368433	C	T	FCGR2A	0.16	0.3148107	0.05609599	2.00E-08	286.52

### Selection of genetic instrumental variables for NAFLD and relevant clinical traits

The genetic IVs of NAFLD and relevant clinical traits were obtained from the GWAS summary statistics. To screen for eligible genetic IVs that met the MR assumptions, a series of quality control measures were performed. Firstly, SNPs needed to reach genome-wide significance with a value of *p* <5 × 10^−8^. Secondly, a linkage disequilibrium (LD) clumping algorithm with R2 < 0.001, window size = 10,000 kb, and value of *p* <5 × 10^−8^ was applied to exclude SNPs that were in strong LD. Finally, to ensure that the effect alleles belonged to the same allele, the exposure and outcome datasets were harmonized to eliminate SNPs with intermediate allele frequencies and ambiguous SNPs with nonconcordant alleles. After these rigorous selections, these SNPs were used as the IVs for subsequent analysis.

### Statistical analysis and data visualization

All statistical analyses and data visualization were performed using the R programming software (R4.1.2).^2^ The Wald ratio and Inverse Variance Weighted (IVW) methods (Burgess et al., 2013) were provided by the “TwoSampleMR” R package (Version 0.5.6). A two-sided value of *p* <0.05 was considered significant. For MR analyses with two more IVs, two complementary approaches, MR-Egger and Weighted-median, were used to ensure the robustness of the analysis. Forest plots were generated using the “forestplot” R package (Version 2.0.1).

## Results

### Causal effects of *Helicobacter pylori* infection on NAFLD

**Figure 2:**
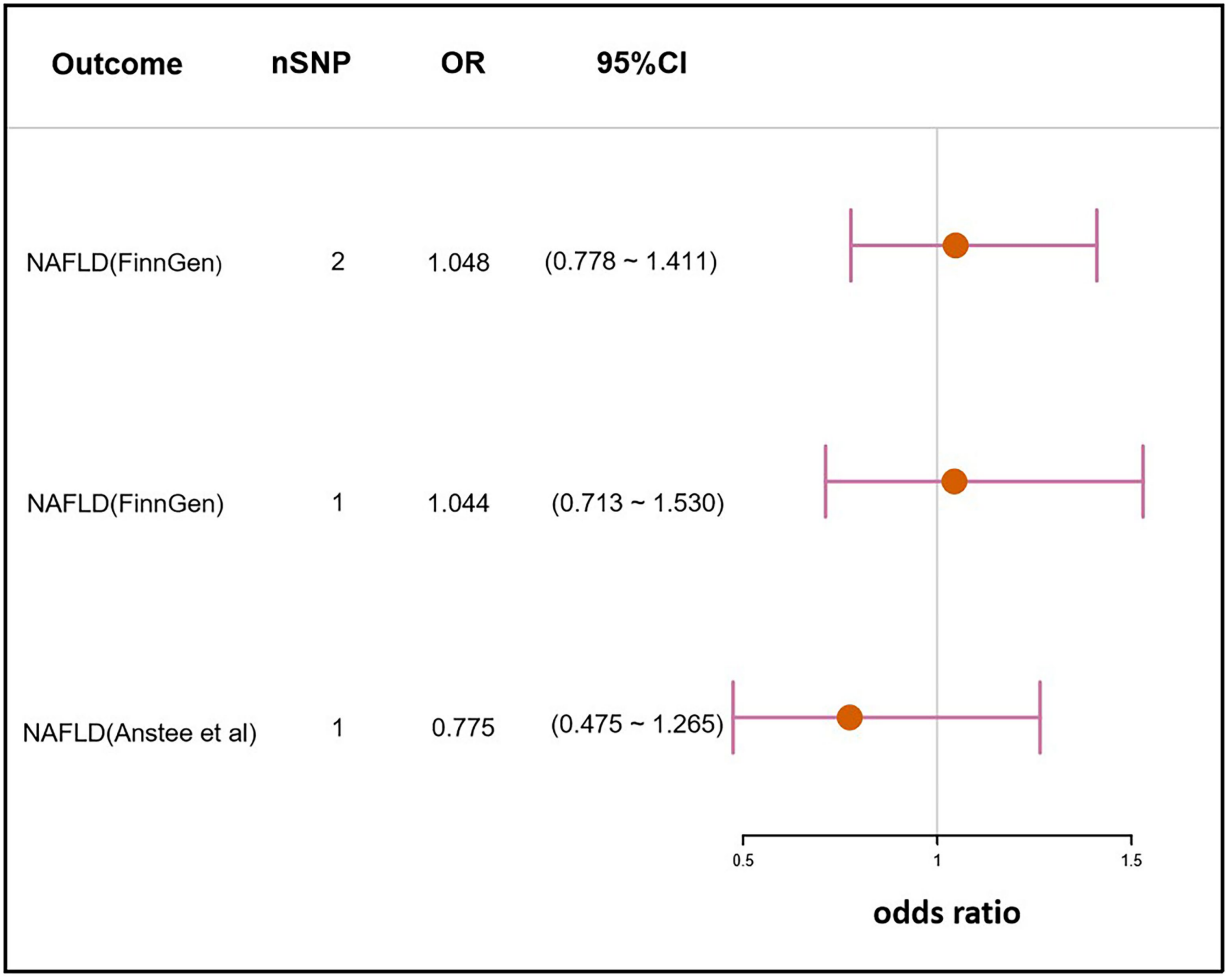
Mendelian randomization result of the effect of *H. pylori* infection on NAFLD. nSNP, the number of SNPs used in the analysis, and the SNP rs10004195 was used if nSNP = 1. The SNPs rs10004195 and rs368433 were used if nSNP = 2. OR, the odds ratio. 95% CI, 95% confidence interval.

The SNP rs10004195 (T > A), a missense variant, and the SNP rs368433 (T > C), an intron variant, were used in the MR analysis working as IVs. Their F statistics were 263.67 and 143.22, respectively, much greater than 30. All genetic associations were aligned to the allele that increases the *H. pylori* seropositivity (Mayerle et al., 2013).Genetically predicted *H. pylori* infection showed no association with NAFLD in the FinnGen GWAS under the IVW method [odds ratio (OR), 1.048; 95% confidence interval (CI), 0.778–1.411; value of *p* =0.759]. Similar results were obtained when only using SNP rs10004195 as IVs and the Wald ratio method (OR, 1.044; 95% CI, 0.713–1.530; value of *p* = 0.824). As SNP rs368433 could not be found in the GWAS summary statistics, only rs10004195 was used as an IV for the MR analysis in the GWAS conducted by Anstee et al. (2020). Additionally, the result was not significant using the Wald ratio method (OR, 0.775; 95% CI, 0.475–1.265; value of *p* = 0.308; Figure 2).

### Causal effects of NAFLD on *Helicobacter pylori* infection

**Figure 3:**
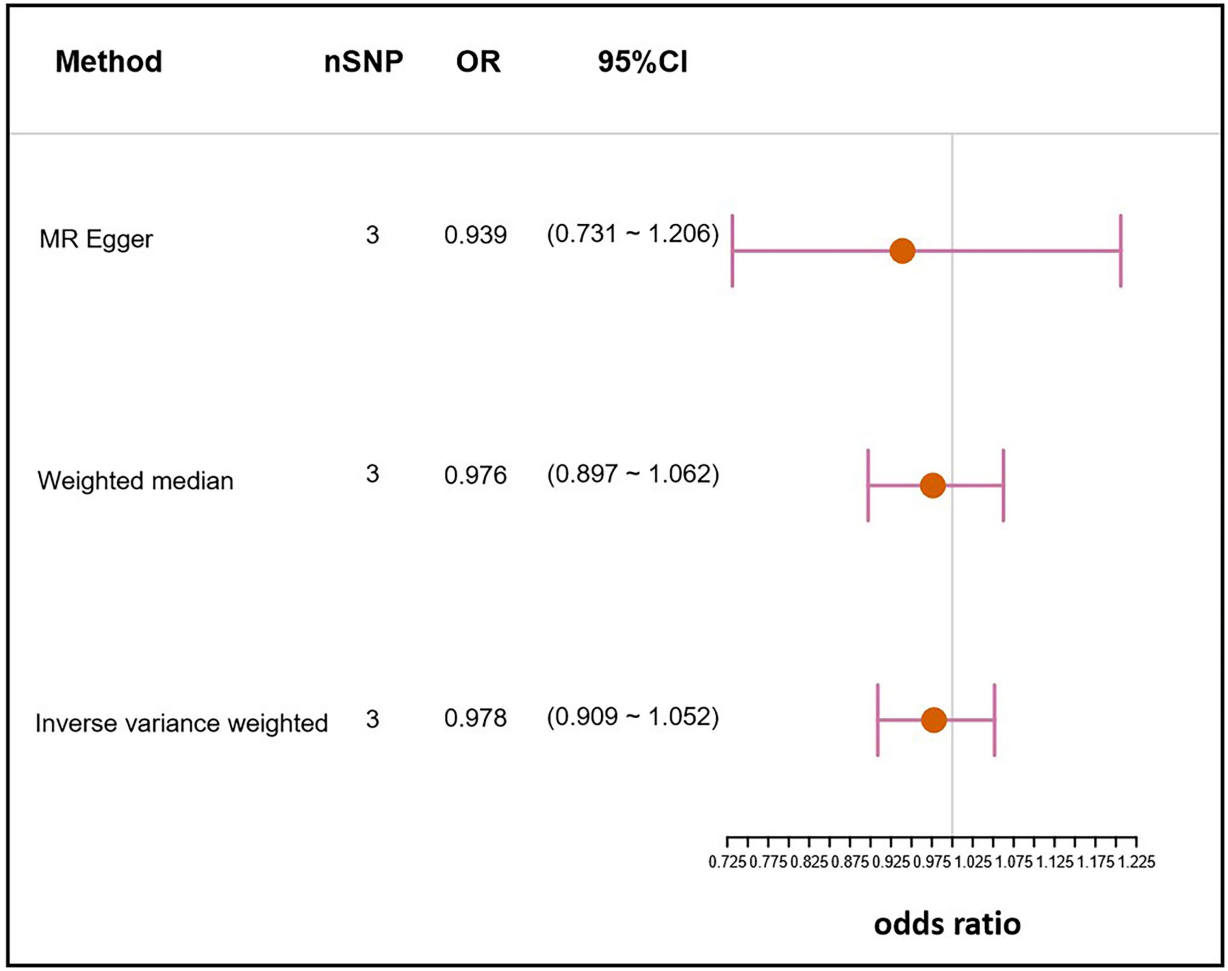
Mendelian randomization result of the effect of NAFLD on *H. pylori* infection. nSNP, the number of SNPs used in the analysis. OR, the odds ratio. 95% CI, 95% confidence interval.

The IVs of NAFLD were identified from the largest NAFLD GWAS conducted by Anstee et al. (2020). A total of 3 SNPs, rs738409, rs13118664, and rs17216588, were selected. The SNP rs738409 (C > G) is a missense variant of the patatin like phospholipase domain containing 3 (PNPLA3) gene which has been confirmed to be associated with the risk of NAFLD in multiple GWASs (Chambers et al., 2011; Sookoian and Pirola, 2011). The SNP rs13118664 is an intron variant of the hydroxysteroid 17-beta dehydrogenase 13 (HSD17B13) gene, and its variant has been reported to be associated with a lower risk of NAFLD and incidences of adverse liver outcomes (Ting et al., 2021; Hudert et al., 2022). Unfortunately, currently no complete information or related publications exist on the SNP rs17216588 (C > T). The F statistic of each SNP was greater than the empirical threshold of 30, indicating less bias caused by weak instruments (Table 3). Genetically predicted NAFLD showed no association with *H. pylori* infection under the IVW method (OR,0.978;95% CI, 0.909–1.052; value of *p* = 0.543). Similar results were obtained using the Weighted-median method and MR-Egger method (Weighted-median OR, 0.976; 95% CI, 0.897–1.062; value of *p*: 0.578; MR-Egger OR, 0.939; 95% CI, 0731–1.206; value of *p* = 0.709; Figure 3).

**Table 3:** Instrumental SNPs of NAFLD and F statistics.

Instrumental SNP	Effect allele	Other allele	Gene	EAF	BETA	SE	*P*	*F*
rs13118664	T	A	HSD17B13	0.21	−0.301645779	0.053181595	1.41E-08	865.14
rs17216588	T	C	–	0.15	0.477475644	0.063805777	7.24E-14	150.78
rs738409	G	C	PNPLA3	0.07	0.602675277	0.040715726	1.45E-49	191.29

“—” stands for not reported.

### Causal effects of *Helicobacter pylori* infection on the relevant clinical traits of NAFLD

**Figure 4:**
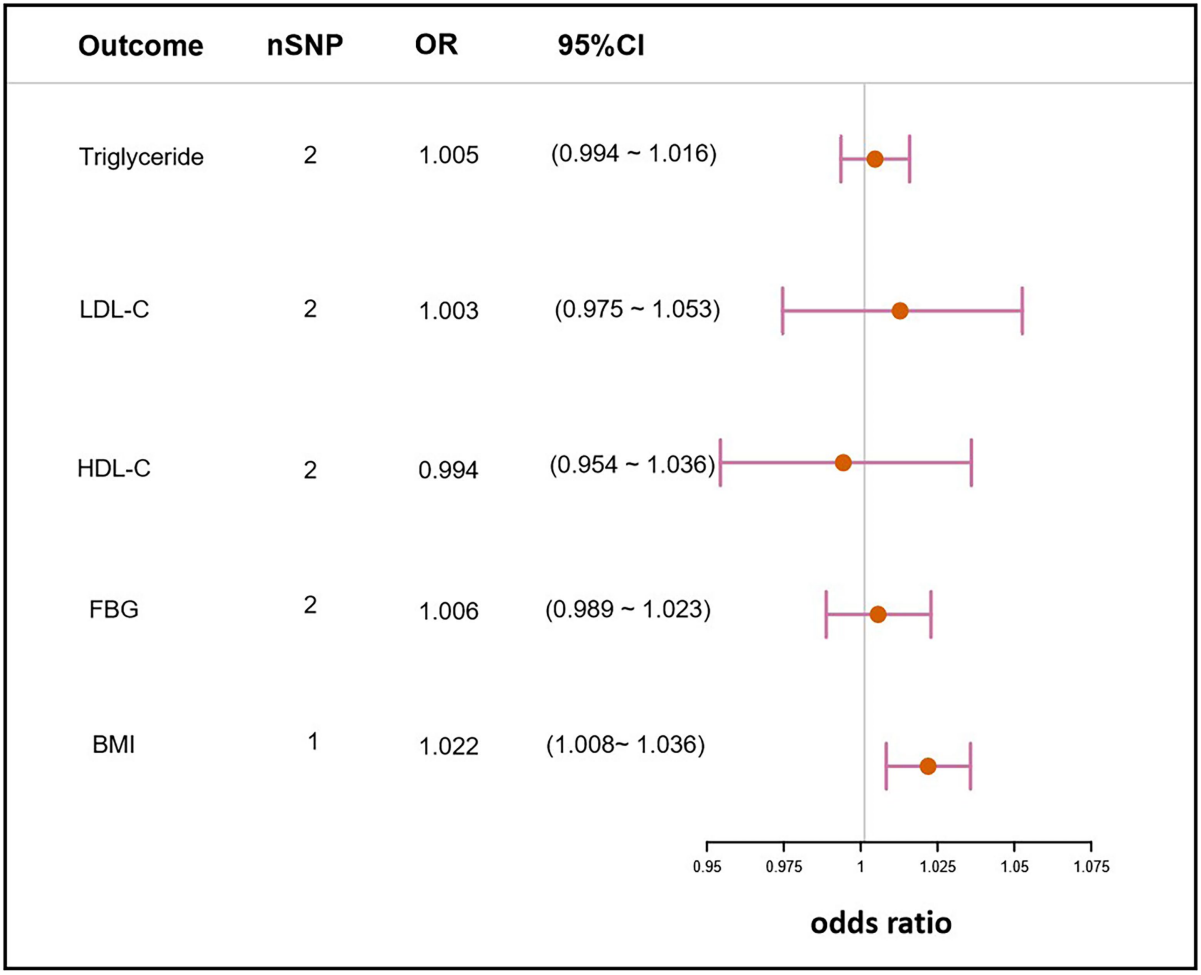
Mendelian randomization result of the effect of *H. pylori* infection on clinical traits related to NAFLD. nSNP, the number of SNPs used in the analysis and the SNP rs10004195 was used if nSNP = 1. The SNPs rs10004195 and rs368433 were used if nSNP = 2. LDL-C, low density lipoprotein cholesterol; HDL-C, high density lipoprotein cholesterol; FBG, fasting blood glucose; BMI, body mass index; OR, the odds ratio. 95% CI, 95% confidence interval.

MR analyses were further performed to examine the causal association between *H. pylori* infection and common NAFLD clinical traits. These clinical traits, including lipidemic, glycemic, and obesity, have been broadly reported to be associated with NAFLD. The analysis revealed that *H. pylori* infection had no causal effect on triglyceride (OR, 1.005; 95% CI, 0.994–1.016; value of *p* = 0.409), LDL-C (OR, 1.003; 95% CI, 0.975–1.053; value of *p* = 0.514), HDL-C (OR,0.994; 95% CI, 0.954–1.036; value of *p* = 0.788), or FBG (OR, 1.006; 95% CI, 0.989–1.023; value of *p* = 0.510). However, there was a statistical significance on BMI (OR, 1.022; 95% CI, 1.008–1.036; value of *p* = 1.47 × 10^−3^), which suggested that *H. pylori* infection could cause the increase in BMI (Figure 4).

### Causal effects of the clinical traits of NAFLD on *Helicobacter pylori* infection

**Figure 5:**
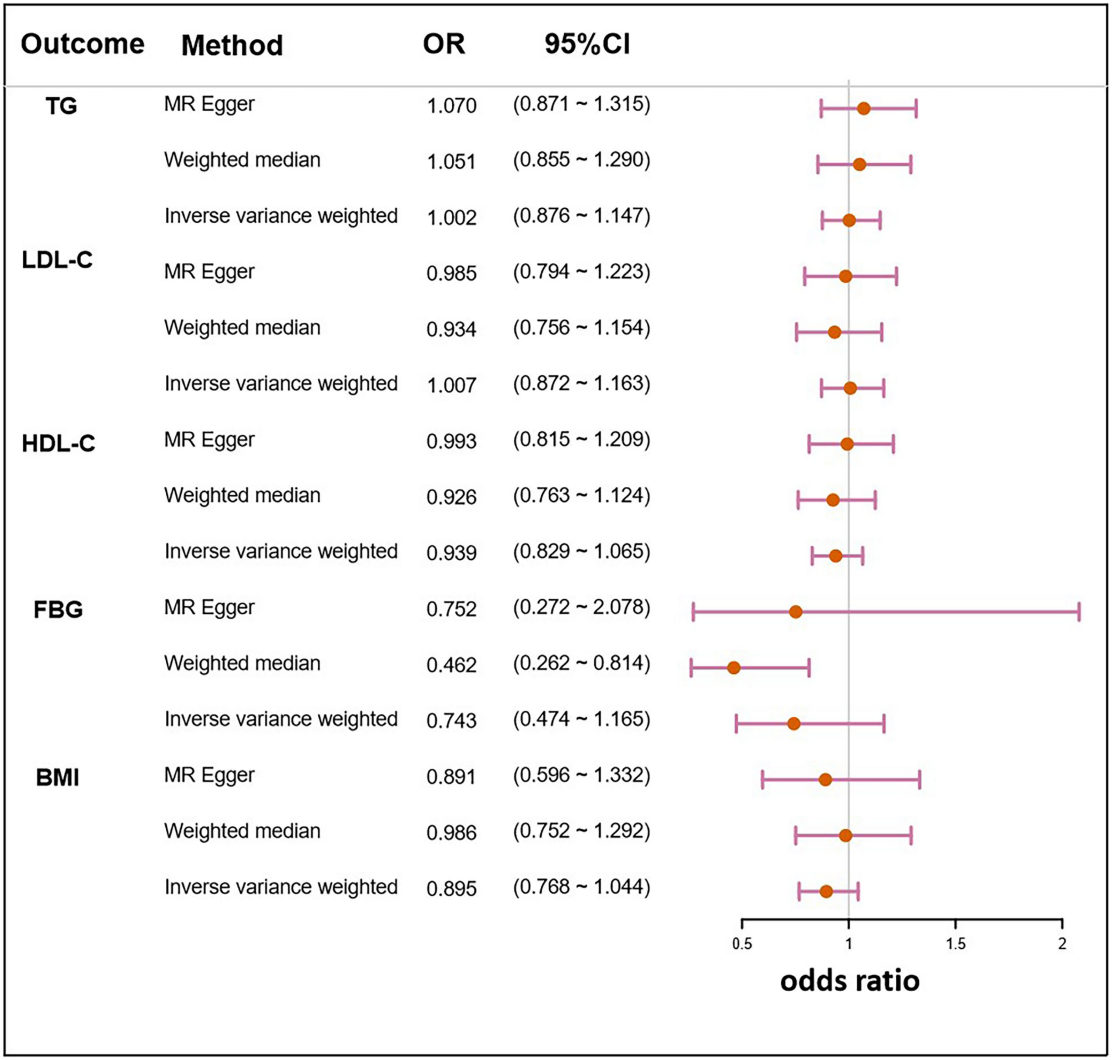
Mendelian randomization result of the effect of clinical traits related to NAFLD on *H. pylori* infection. LDL-C, low density lipoprotein cholesterol; HDL-C, high density lipoprotein cholesterol; FBG, fasting blood glucose; BMI, body mass index; OR, the odds ratio. 95% CI, 95% confidence interval.

The IVs of NAFLD traits were identified from their respective GWAS summary data. In the MR analyses of clinical traits, no genetic evidence of causal effects on *H. pylori* infection was identified (Table 3). Two other methods, the weighted median and MR-Egger also did not demonstrate significant causal effects on *H. pylori* infection (Figure 5).

## Discussion

In the present study, we tried to explore the association between *H. pylori* infection and NAFLD risk by the bidirectional MR method, which is a natural RCT, using publicly shared large-scale GWAS data. Our results showed that there was no significant causal relationship between *H. pylori* infection and NAFLD risk.

There was inconsistent evidence to show the causal effect of *H. pylori* infection on the risk of NAFLD. A cross-sectional study performed on northern Chinese patients reported that *H. pylori* infection is independently associated with an increased risk of NAFLD (OR, 1.27; 95%CI, 1.07–1.50; Jiang et al., 2019). Two cohort studies performed in Egypt and Korea have also reached similar conclusions (Kim et al., 2017; Abdel-Razik et al., 2018). In addition, another cross-sectional study showed that the prevalence of *H*. *pylori* infection was higher in the NAFLD group than in the control group (41.25% vs. 36.85%, value of *p* < 0.001; Yu et al., 2018), which indicated that NAFLD incidences were also related to *H. pylori* infection risk. Nevertheless, the causal relationship between them remains controversial because of the studies with opposing findings. One case–control study performed in Guatemala reported that *H. pylori* infection was not related to NAFLD or other metabolic abnormalities (Alvarez et al., 2020). Other cross-sectional studies performed in Japan and Korea showed that the prevalence of *H. pylori* was not a risk factor for NAFLD (Okushin et al., 2015; Han et al., 2021). One European cohort study also described similar conclusions (Wernly et al., 2022). In addition, Lecube et al. conducted a study to investigate the relationship between *H. pylori* and NAFLD in 93 subjects with both gastric and liver biopsies, and the results showed that *H. pylori* infection did not seem to be associated with abnormal metabolism or advanced degrees of NAFLD (Lecube et al., 2016). Recently, several cross-sectional studies performed in China also found that *H. pylori* infection did not appear to increase the prevalence rate of, or to be associated with, or be a risk factor for, NAFLD (Cai et al., 2018; Fan et al., 2018; Liu et al., 2021; Wang et al., 2022).

The controversy between *H. pylori* infection and the risk of NAFLD could be attributed to several reasons. Firstly, these studies were all observational studies, lacking randomized, prospective, and blinded methods. The discrepancy among the findings is likely due to the limitations of the observational studies. Secondly, the diagnostic approaches used for *H. pylori* infection and NAFLD were different. According to World Gastroenterology Organization (WGO) Global Guidelines, urea breath tests (UBTs) are the best recommended noninvasive test for *H. pylori* infection (Katelaris et al., 2021). In some studies, *H. pylori* infection was determined by serum or fetal IgG antibodies against *H. pylori,* which were not accurate enough (Kim et al., 2017; Abdel-Razik et al., 2018; Abo-Amer et al., 2020; Alvarez et al., 2020). Liver biopsy is the gold standard method for NAFLD diagnosis; however, most of the studies used ultrasonography or FibroScan to diagnose NAFLD because of their noninvasiveness and security. Thirdly, the prevalence of *H. pylori* infection is distinct in various geographical regions, which may influence the effect of *H. pylori* infection on NAFLD. According to a meta-analysis study conducted by Hooi et al. (2017), the prevalence of *H. pylori* is approximately, 79.1% in Africa, whereas 54.7% in Asia, 47% in Europe, and 37.1% in North America (Hooi et al., 2017). A subgroup analysis of one meta-analysis study discovered that *H. pylori* infection was only associated with NAFLD risk in Asia. These differences could be attributed to the different ethnic group’s lifestyles, dietary patterns, and socioeconomic statuses.

The current study also explored the correlation between *H. polyri* infection and the relevant metabolic characteristics of NAFLD. The results showed that there was no significant correlation between *H. pylori* infection and the levels of TG, LDL-C, HDL-C, or FBG. Epidemiological studies have been performed to explore the association between *H. pylori* infection and lipid profiles, some of which have reported a significant correlation between *H. pylori* infection and elevated lipid levels (Kim et al., 2011; Shimamoto et al., 2020). However, the results are controversial. Several studies reported that no significant differences in serum lipid levels were found between the *H. pylori* positive group and the *H. pylori* negative group, and the lipid levels also did not change significantly following *H. pylori* eradication (Elizalde et al., 2002; Akbas et al., 2010; Watanabe et al., 2021). This is probably due to differences in the general characteristics of the study populations and the lack of control for confounding factors related to lipid profiles. Therefore, extracting conclusions from observational studies is a difficult task. The MR study allows for a more robust and substantiated conclusion with the advantages of being free of these issues. It is worth noting that a causal effect of *H. pylori* infection on an increase in BMI was found in our study, without a reverse result. A study performed on Danish adults reported that the seroprevalence of *H. pylori* infection is increased in people with a high BMI (Rosenstock et al., 2000). Nevertheless, *H. pylori* may be the cause of the increase in BMI, and we cannot discount the possibility of reverse causation.

The identification of mechanisms underlying NAFLD and the uncovering of novel therapeutic targets are of high priority while the treatment for *H. pylori* infection is easy and relatively inexpensive. Thus, this concern has received considerable attention (Cheng et al., 2017). Our study is the first study to reveal the causal relationship between *H. pylori* infection and NAFLD, and a bidirectional MR analysis was carried out to clarify the causation. The study could increase the recognition of pathogenic factors of NAFLD from the perspective of systems biology. However, there are some limitations in our study. Firstly, the diagnosis of *H. pylori* infection was based on serological testing in the GWAS data, which may have biased on the detection of *H. pylori* infection. Secondly, the dataset we used only included the European population. Although using a single European population to investigate the causal relationship can minimize population stratification bias, it might not be generalizable to other populations. Thirdly, two IVs of *H. pylori* infection were used in estimating the causal effect, which could guarantee the power of the MR study; however, the strict selection of IVs may cause a false negative result. Fourthly, the relationships between the risk factors for *H. pylori* infection such as Vitamin B12 deficiency, iron deficiency anemia, and primary immune thrombocytopenia, and NAFLD were not defined due to a lack of related GWAS data. Fifthly, the relationships between *H. pylori* infection and the fibrosis indexes were not defined also due to a lack of related GWAS data. In addition, the proportion of NAFLD cases was not large enough which might also reduce the statistical power.

## Conclusion

Our MR study did not find a causal link between *H. pylori* infection and NAFLD risk, suggesting that eradication or prevention of *H. pylori* infection might not benefit for NAFLD and vice versa. However, because of limitations in the serological diagnosis of *H. pylori,* further GWAS based on histological diagnoses and more MR studies may be needed to assess the relationship between *H. pylori* infection and NAFLD.

## Data availability statement

The original contributions presented in the study are included in the article/Supplementary material, further inquiries can be directed to the corresponding author.

## Ethics statement

Written informed consent was obtained from the individual(s) for the publication of any potentially identifiable images or data included in this article.

## Author contributions

YL analyzed the study data and wrote the manuscript. YL and HX performed the MR analyses and analyzed the data. ZZ and YD assisted with data collection and the analysis. XW and JN conceived the study, supervised the research, and edited the manuscript. All authors contributed to the article and approved the submitted version.

## Funding

This work was sponsored by the National Natural Science Foundation of China (grants no. 81970519), Natural Science Foundation of Jilin Province (20210101446JC), Program of Jilin Health Talents (JLSWSRCZX2021), and Program of Jilin Educational Department (JJKH20221085KJ).

## Conflict of interest

The authors declare that the research was conducted in the absence of any commercial or financial relationships that could be construed as a potential conflict of interest.

## Publisher’s note

All claims expressed in this article are solely those of the authors and do not necessarily represent those of their affiliated organizations, or those of the publisher, the editors and the reviewers. Any product that may be evaluated in this article, or claim that may be made by its manufacturer, is not guaranteed or endorsed by the publisher.

## Footnotes

^1^ https://www.bristol.ac.uk/integrative-epidemiology/

^2^ https://www.rproject.org/
